# Understanding the Detachment–Strain Relationship: A Two-Wave Mediational Model

**DOI:** 10.3390/ejihpe15120246

**Published:** 2025-11-29

**Authors:** Chiara Consiglio, Nicoletta Massa, Ferdinando Paolo Santarpia, Cristiano Violani

**Affiliations:** Department of Psychology, Sapienza University of Rome, 00185 Rome, Italy; nicoletta.massa@uniroma1.it (N.M.); ferdinandopaolo.santarpia@uniroma1.it (F.P.S.); cristiano.violani@uniroma1.it (C.V.)

**Keywords:** healthcare professionals, stressor–detachment model, psychological detachment, spillover, exhaustion

## Abstract

Background: Healthcare professionals are highly exposed to work-related stressors, which increases their vulnerability to exhaustion, a key dimension of burnout. Psychological detachment, defined as the ability to mentally disengage from work during off-job time, has been identified as a key recovery mechanism. However, the processes linking detachment to exhaustion over time remain underexplored. Methods: This study integrated the Conservation of Resources theory and the Stressor–Recovery Model to test a two-wave longitudinal model, in which negative work–home spillover mediates the relationship between psychological detachment and exhaustion. The reverse pathways were also examined. Data were collected from 258 healthcare professionals at an Italian hospital, who completed self-report questionnaires at two time points over a two-year period. Structural equation modeling was employed to test the hypothesized relationships. Results: Psychological detachment at Time 1 predicted lower spillover at Time 2, which in turn was associated with lower exhaustion, supporting a full mediation model. Additionally, spillover at Time 1 negatively predicted detachment at Time 2, suggesting the existence of a potential loss cycle, while reverse effects from exhaustion to spillover or detachment were not supported. Conclusions: These findings highlight the crucial role of psychological detachment in preventing long-term exhaustion among healthcare professionals by reducing work–home spillover, providing valuable insights for interventions that promote recovery processes.

## 1. Introduction

The healthcare sector is widely recognized as a work environment characterized by high emotional, cognitive, and organizational demands (Maslach & Leiter, 2000). Healthcare professionals are continuously exposed to critical situations, emotionally intense interactions, and high workloads, making them especially vulnerable to work-related strain and ill health (Shirom & Melamed, 2005; Maslach, 2003; Gilin Oore et al., 2010). These stressors can significantly impact mental health (Laschinger & Grau, 2012; Laschinger et al., 2010), leading to organizational costs such as employee turnover, reduced performance (Bourdeanu et al., 2020; Lee et al., 2020; Gandi et al., 2011; Zhang et al., 2023), and contributing to job burnout (Maslach, 2003; Maslach et al., 2001; Balducci et al., 2020; Bridgeman et al., 2018). Notably, burnout is conceptualized as a psychological syndrome resulting from chronic exposure to work-related stress, with exhaustion representing its core dimension (i.e., feelings of being emotionally overextended and depleted of one’s emotional and physical resources) (Maslach, 2003; Maslach & Leiter, 2000; Maslach & Jackson, 1981). Given these risks, identifying mechanisms that help employees recuperate their mental and physical resources after work is essential.

Recovery from work stress has thus emerged as a key process for preserving employees’ well-being (Sonnentag et al., 2010), alleviating fatigue, and restoring resources during off-job time. Empirical evidence has consistently shown that recovery experiences (i.e., psychological detachment, relaxation, mastery, and control) are associated with improved well-being, health, and even job performance (Sonnentag & Bayer, 2005). Among recovery experiences, psychological detachment—defined as mentally disconnecting from work and work-related matters during off-hours (Etzion et al., 1998)—has been described as a core mechanism within the Stressor–Detachment Model (Sonnentag & Fritz, 2007). To date, research on psychological detachment has primarily focused on its role in attenuating the impact of job stressors on strain (e.g., Moreno-Jiménez et al., 2009) or on its direct association with well-being outcomes (e.g., Headrick et al., 2023; Sonnentag & Fritz, 2007; Sonnentag et al., 2010; Sonnentag & Fritz, 2015).

Parallel literature highlights the concept of negative work–home spillover; namely, the cognitive and emotional intrusion of work into private life, which impairs recovery and fosters work–life conflict (Martinez-Corts et al., 2015). We argue that psychological detachment and spillover represent distinct yet interconnected processes: the former reflects an intentional strategy to restore mental energy, whereas the latter represents an involuntary continuation of work-related activation into non-work time. Despite their conceptual proximity, their relationship remains poorly understood.

In line with the Stressor–Detachment model (Sonnentag & Fritz, 2007), we claim that psychological detachment reduces exhaustion by inhibiting the cognitive activation of work-related memories during off-job time. This mechanism underscores the role of detachment not only as a recovery experience, but also as a potential cognitive filter that limits the resurgence of emotionally activating work episodes.

Since most previous studies rely on cross-sectional (e.g., Safstrom & Hartig, 2013; Sonnentag et al., 2010; Sonnentag & Fritz, 2015) or short-term longitudinal designs (Sonnentag et al., 2017), research has yet to clarify whether psychological detachment reduces exhaustion by preventing spillover, and how these dynamics unfold over more extended time frames (i.e., years). The two-year lag, therefore, aligns with studies that have investigated recovery effects across medium-to-long time frames (4–24 months; Sonnentag et al., 2022) and allows us to explore whether detachment contributes to shaping enduring associations with employee well-being.

Moreover, potential reverse relationships have been largely overlooked (Sonnentag et al., 2022). Drawing on the Conservation of Resources (COR) theory (Hobfoll, 1989), we propose that resource loss may trigger maladaptive cycles in which exhaustion itself impairs the use of recovery strategies like detachment, by activating ruminative processes that increase the likelihood of mentally recalling stressful experiences that occurred during the workday once at home (i.e., spillover). Understanding the directionality and reciprocal dynamics among these variables is crucial for a more comprehensive model of strain regulation and recovery.

Based on these considerations, the present study aims to reach two main goals:

- To explore whether psychological detachment reduces exhaustion over time by limiting negative work–home spillover
- To examine whether exhaustion and negative spillover, in turn, prospectively impair psychological detachment

Overall, this research makes four main contributions. First, it advances the literature on recovery strategies by integrating two previously separate lines of research—detachment and spillover—within a unified, process-based framework. Second, it introduces a long-term temporal perspective (i.e., two years), addressing the need for more longitudinal studies with extended time lags (Sonnentag et al., 2017, 2022). Third, it identifies spillover as the psychological mechanism through which detachment exerts its effects on exhaustion, thereby contributing to the further refinement of the Stressor–Detachment Model. Fourth, it contributes to a better understanding of the loss cycles described in COR theory by explicitly testing reverse effects; namely, whether exhaustion undermines recovery potential via increased spillover and reduced detachment.

All in all, the proposed framework makes explicit how the Conservation of Resources theory and the Stressor–Detachment Model complement one another: COR clarifies how resource loss and preservation processes unfold over time, while the Stressor–Detachment Model specifies psychological detachment as a key recovery mechanism. Integrating these perspectives offers a more comprehensive perspective of recovery and strain regulation over time.

All in all, the proposed framework clarifies how the Conservation of Resources theory and the Stressor–Detachment Model complement each other. While the Stressor–Detachment Model identifies psychological detachment as a key recovery strategy that preserves personal resources, COR theory explains how resource loss cycles may unfold when the recovery process is impaired. Integrating these perspectives allows us to conceptualize spillover as the mechanism through which resource depletion unfolds over time, offering a more comprehensive understanding of recovery and strain regulation.

### 1.1. The Recovery Process from Psychological Detachment to Reduced Exhaustion: The Role of Spillover

As outlined in the literature, the more a person psychologically detaches from stressful events happening in the work context, the less adverse outcomes on and off the job over time they will experience (Sonnentag & Fritz, 2015; Sonnentag et al., 2010, 2017, 2022; Schulz et al., 2021). In particular, the relationship between psychological detachment and exhaustion has been investigated primarily through cross-sectional studies (e.g., Safstrom & Hartig, 2013; Sonnentag et al., 2010) and meta-analysis (Headrick et al., 2023), with few longitudinal contributions (e.g., Schulz et al., 2021; Muhamad Nasharudin et al., 2020). For instance, in a diary study, Sonnentag and Bayer (2005) found that psychological detachment from work-related topics once off the job was related to less fatigue at bedtime. In a two-wave study, Muhamad Nasharudin et al. (2020) demonstrated that psychological detachment resulted in less exhaustion four months later, highlighting the importance of this recovery strategy in promoting individual well-being. Although additional studies support this direction (e.g., Davidson et al., 2010), a lack of homogeneity in results on this issue remains, as well as a need for a comprehensive understanding of the time lag necessary for detachment outcomes to be meaningful. In this regard, Sonnentag et al. (2022) outlined a stronger recovery effect in the short term than in the long term. This result suggests that different mechanisms may be involved in the recovery process.

To address this gap, we propose that the beneficial effects of detachment on strain operate through its capacity to reduce work–home spillover—a psychological process whereby thoughts and emotions associated with the work context intrude into the private domain—over time. According to Sonnentag and Binnewies (2013), higher levels of psychological detachment at the end of the working day are associated with a reduction in the spillover of what was experienced in the workplace once the job is over. Indeed, psychological detachment functions as an intentional mental strategy aimed at ceasing work-related thoughts and emotions once off the job, as well as to avoid getting caught up in work tasks once one leaves work (Sonnentag & Bayer, 2005). Specifically, during the transition from one psychosocial role to another—i.e., the transition from the work domain to the off-the-job domain—the psychological detachment from what happened in the workplace prevents its repercussion in the person’s private life (Edwards & Rothbard, 2000). Accordingly, Derks and Bakker (2014) demonstrated that daily levels of psychological detachment hinder spillover processes, preventing the exhaustion caused by job demands from affecting people’s well-being once at home. Furthermore, the diary study by Gombert et al. (2018) demonstrated the buffer effect promoted by psychological detachment on ego-depletion at home, highlighting how this recovery strategy prevents job demands from continuing to affect well-being through active mental recall (i.e., spillover). This is particularly relevant for healthcare professionals, for whom the emotional investment required in patient care may continue to burden them in their personal lives, consequently exposing them to emotional distress (Van Mol et al., 2015; Rushton et al., 2015) and decreased sleep quality (Cho et al., 2024). Accordingly, we claim that mentally disengaging from work will stem the risk of mentally re-experiencing episodes that occurred at work, reducing their emotional significance and preventing the spillover process from being activated once at home. Therefore, we formulated the following hypothesis:

**Hypothesis** **1 (H1):** *Psychological detachment at T1 is negatively related with spillover at T2.*

Previous studies investigated how the perceived cognitive and affective interference resulting from the work environment within the household (i.e., work–family conflict) negatively affects people’s lives (Ford et al., 2007; Michel et al., 2011). For instance, a meta-analysis (Alarcón, 2011) suggests that spillover and the consequential role conflict are associated with burnout. Querstret and Cropley (2012) found that work-related rumination is related to increased fatigue. Additionally, other interesting evidence highlights the cognitive loop through which recursive thinking and worry contribute to impaired well-being (e.g., Kinnunen et al., 2017; Firoozabadi et al., 2018). Indeed, continuing to think about unpleasant work-related events at the end of the working day will keep the person in a state of ‘cognitive perseverance’ (Ottaviani et al., 2016), leading to a sustained activation even in the absence of the original job demand, which is thus experienced again through its mental representation (Meurs & Perrewé, 2011; Ursin & Eriksen, 2010). Hence, the incessant use of cognitive resources on work issues during non-work time, such that it creates conflict and stress, likely leaves individuals mentally exhausted, depleted, and without the personal resources required to reinvest during work time (Bakker et al., 2014). From this perspective, we argue that spillover represents a mechanism that extends the depletion of resources within the private domain (Hobfoll, 1991; Hobfoll et al., 2018), recreating a mental connection with job-related issues and gradually draining the person’s resources.

Based on this, we hypothesized that:

**Hypothesis** **2 (H2):** *Spillover at T1 is positively related with exhaustion at T2.*

However, suppose healthcare professionals can contain the intrusiveness of thoughts associated with work experiences in their private lives during non-work time. In that case, they may be better able to prevent the mental and emotional recurrence of work-related experiences in their personal lives, thereby protecting themselves from prolonged psychological depletion. In this sense, psychological detachment operates as a cognitive gatekeeper that limits the activation and reactivation of emotionally charged work episodes during off-job time (Sonnentag & Fritz, 2015; Sonnentag et al., 2022). Through this mechanism, psychological detachment not only promotes psychological and emotional restoration but also interrupts the ongoing spiral cycle of resource loss (Hobfoll et al., 2018), which would otherwise be perpetuated through spillover-induced depletion of personal resources.

Hence, we propose that the protective effect of psychological detachment on exhaustion occurs indirectly, through its capacity to inhibit spillover and to interrupt the recursive loop of resource loss. Therefore, we hypothesized that:

**Hypothesis** **3 (H3):** *The negative relation between psychological detachment at T1 and exhaustion at T2 is mediated by spillover.*

### 1.2. The Loss Process from Exhaustion to Psychological Detachment: A Recovery Paradox

In the stressor–detachment model, which systematizes empirical evidence on the relationship between detachment and exhaustion, recovery is assumed to temporally precede job strain, exerting a buffering effect that limits its onset (Sonnentag & Fritz, 2015). Although research has suggested an inverse causal relationship between psychological detachment and exhaustion, empirical results remain mixed (Sonnentag et al., 2022). For instance, a 4-week diary study by Sonnentag et al. (2014) found that people who felt more exhausted were less able to detach themselves from work. Schulz et al. (2021) found that a high level of exhaustion can decrease the frequency of psychological detachment six months later. Other studies did not find significant reverse associations over time (e.g., Sianoja et al., 2018; Sonnentag et al., 2010).

The paradoxical nature of the detachment–exhaustion relationship is rooted in a conflict between the need to detach oneself from the demands of a work environment, perceived as excessive and overtaxing, and an actual reduction in the propensity to enact recovery (Sonnentag, 2018). The loss spiral activated by exhaustion has been traced to the cognitive and affective dysregulation experienced by burned-out employees (Sonnentag et al., 2014). Indeed, recent empirical evidence found that burnout is linked with biases in emotional processing, such as increased fixation on sadness/loss-related stimuli and decreased fixation on happiness/pleasure-related stimuli (Bianchi & Laurent, 2015), as well as under-recall of positive stimuli and an over-recall of negative stimuli (Bianchi et al., 2020). In this regard, exhaustion has been extensively associated with enhanced perseverative cognition (e.g., McCarrick et al., 2024) and rumination over job-related distress and its causes (e.g., Sousa & Neves, 2021; Vandevala et al., 2017; Bianchi & Schonfeld, 2016).

Building on these evidences, we propose that the real driver of impaired detachment is increased cognitive and emotional spillover. Accordingly, Sonnentag and Fritz (2015) argued that the tendency of exhausted employees to remain mentally engaged with work-related issues during off-job time may reflect a coping attempt. For instance, if employees cannot cope with chronic stressors occurring during the shift (e.g., frustrating interactions with patients or a lack of procedural clarity in their unit), they may try to reassess these experiences when work demands are absent to come up with solutions. For exhausted individuals, the maladaptive aspect of this strategy may lie in the fact that they lack the resources to generate an alternative, problem-focused thought process (Hobfoll & Freedy, 2017), bounding themselves in a vicious loop revolving around the stressors and one’s strain reactions; e.g., brooding over failures and wondering why they deserved such adverse events (Bianchi & Schonfeld, 2016). Along these lines, we argue that spillover experiences (e.g., recalling work activities and shift hassles during leisure time) may naturally intensify as exhaustion increases, making the stressful dimension of work central (i.e., perceived resource losses or threat of future losses) to a person’s narrative about themselves and their world in the non-work domain. Recalling the Conservation of Resources theory (Hobfoll, 1989) and, in particular, the desperation principle (Hobfoll et al., 2018), we claim that under such conditions, exhausted employees may become cognitively and emotionally entangled in work-related concerns, attempting to regain control. Still, the lack of adaptive resources traps them in a cycle of negative spillover once at home.

Hence, we hypothesized that:

**Hypothesis** **4 (H4):** *Exhaustion at T1 is positively associated with spillover at T2.*

At the same time, a frequent spillover may keep the cognitive and affective cues of the original negative work experience readily retrievable, depleting recovery processes (Sonnentag et al., 2014). Indeed, spillover maintains the salience and accessibility of work-related cognitive and affective cues even during non-work time, thereby sustaining a condition of continued mental activation (Cropley & Zijlstra, 2011; Ottaviani et al., 2016). When such intrusions persist into private life, they tend to foster recursive thinking patterns—such as rumination or worry—that interfere with the cognitive disengagement required for detachment. Importantly, in these cases, psychological detachment becomes more difficult not because of new external stressors, but due to the internal persistence of unresolved cognitive–emotional traces of prior work experiences (Sonnentag & Fritz, 2015; McCarrick et al., 2024)

Therefore, we posit that spillover represents a mechanism through which resource loss extends into the private domain, reactivating work-related concerns and gradually eroding employees’ capacity to recover. From this perspective, we claim that spillover impairs psychological detachment, as it fosters a continuous mental engagement with the work domain that hampers the cognitive disengagement process necessary for recovery. Thus, we hypothesized that:

**Hypothesis** **5 (H5):** *Spillover at T1 is negatively associated with psychological detachment at T2.*

Consequently, spillover could be an explanatory factor in the recovery impairment of the exhausted employee. Faced with chronically overwhelming experiences, the exhausted person might begin to spiral over their present and future losses at work and how to prevent them. In turn, since they lack resources to effectively cope with work activities and stressors, continuing to persevere cognitively with work-related stressors should explain the gradual depletion of their ability to detach psychologically at the end of work shifts. This mechanism can be further understood by recognizing that psychological detachment represents a deliberate effort that requires mental clarity, self-regulation, and emotional distancing. When exhaustion depletes these internal capacities, individuals may find themselves unable to engage in the very strategies that would otherwise restore their balance (Sonnentag, 2018; Sonnentag et al., 2014; Hobfoll & Freedy, 2017). Paradoxically, it is precisely in moments of greatest need for psychological recovery that individuals are least equipped to pursue it. In this context, spillover functions as a bridge between depletion and impaired recovery, sustaining the mental presence of work-related concerns beyond working hours and reinforcing the erosion of boundaries necessary for mental disengagement. Drawing on the Conservation of Resources theory (Hobfoll et al., 2018), this reflects a form of resource loss spiral, in which exhaustion weakens recovery potential by fueling spillover, which in turn obstructs detachment.

Therefore, we hypothesized that:

**Hypothesis** **6 (H6):** *The negative relationship between exhaustion at T1 and psychological detachment at T2 is mediated by spillover.*

## 2. Materials and Methods

### 2.1. Sample and Procedure

The study was conducted using an anonymous online self-report questionnaire administered via the Qualtrics platform. Data collection occurred at two time points spaced two years apart. Participants were employees of a large Italian university hospital. To enable the longitudinal matching of responses while preserving anonymity, each participant created a unique identification code known only to the person. Before participation, informed consent was obtained after providing participants with complete information on the study’s objectives, the voluntary nature of their involvement, and the confidentiality of their data. The study adhered to the ethical principles outlined in the Declaration of Helsinki and complied with current data protection regulations.

The survey was distributed to all members of the participating healthcare organization, including medical, nursing, and administrative staff. All participants were salaried employees working under standard contractual arrangements. No exclusion criteria were applied, except for employment within the organization at the time of data collection. At Time 1, 719 employees completed the questionnaire (response rate of 40%), and at Time 2, 895 participated (response rate of 51%), resulting in a final matched sample of 258 respondents. Missing or incomplete cases across waves were excluded from the longitudinal analyses.

The sample comprised 258 employees, with 65.4% being females and 34.6% being males. Regarding age, 42.3% of participants ranged from 46 to 55 years old, and 39.6% ranged from 36 to 45 years old. Fewer participants were lower than 35 years old (4.2%) or older than 55 years (13.5%). With respect to professional role, 45.8% of participants were nurses, 29.2% were physicians, 17.7% belonged to the administrative staff, and 2.7% were healthcare support staff. The remaining 4.6% held roles not classifiable into the above categories. Organizational tenure ranged from 1 to 30 years (M = 11.46, SD = 3.81). Regarding caregiving at home (for minor children or non-autonomous individuals), the majority of the sample did not care for others (59.6%), while the remaining participants cared for others (40.0%).

### 2.2. Measures

#### 2.2.1. Psychological Detachment

Psychological Detachment (3 items; Sonnentag & Fritz, 2007) measured the degree to which the employee mentally disengages from work (e.g., ‘‘I forget about work”; “I get a break from the demands of my work”). All items were introduced by an incipit that reads, “At the end of my working hours...” Participants rated the frequency of psychological detachment on a seven-point Likert scale, ranging from 1 (“never”) to 7 (“always”). The reliabilities of the scale were α = 0.91 at T1 and α = 0.94 at T2.

#### 2.2.2. Spillover

Spillover (4 items; adapted from van Der Doef & Maes, 1999) assessed perceptions of cognitive and affective interferences arising from the working context at home (e.g., “Because of my work, I often find it difficult to fulfil my domestic obligations”; “I carry the tensions of work in my free time at home”). The Italian translation followed a standard translation–back-translation procedure, and previous research has confirmed its factorial validity in Italian occupational samples. Higher scores reflect a stronger tendency for work-related thoughts or emotions to intrude into private life. Participants rated their level of agreement on a four-point Likert scale, ranging from 1 (“not at all”) to 4 (“completely”). The reliabilities of the scale were α = 0.79 at T1 and α = 0.85 at T2.

#### 2.2.3. Exhaustion

Exhaustion (5 items from the MBI-GS; Schaufeli et al., 1996, Italian adaptation by Borgogni et al., 2005) assessed feelings of being over-extended and drained from energetic resources about one’s work (e.g., “‘I feel used up at the end of the workday”; “I feel emotionally drained from my work”). Participants rated the frequency of exhaustion on a seven-point Likert scale ranging from 1 (“never”) to 7 (“always”). The reliabilities of the scale were α = 0.92 at T1 and α = 0.91 at T2.

### 2.3. Modeling Strategies

In testing our theoretical model, we employed an autoregressive, cross-lagged design, which is currently recognized as one of the strongest and least biased designs for assessing mediation using two time points (Cole & Maxwell, 2003; Maxwell & Cole, 2007). Under this framework, the product of the coefficients associated with (1) the cross-lagged relationship between psychological detachment at T1 with spillover at T2, and (2) the cross-lagged relationship of spillover at T1 with exhaustion at T2 provides an estimate of the partial regression coefficient associated with the mediated effect from psychological detachment to exhaustion over time. Autoregressive paths were included so that each cross-lagged path takes into account the stability of the variables and thus more reliable estimates of the parameters are obtained. Indeed, mediational processes may be investigated with two waves of data under the assumption that the structure of the relationships among variables is the same over time (i.e., stationarity; Cole & Maxwell, 2003). Reciprocal cross-lagged paths (i.e., psychological detachment and spillover at T2 on exhaustion at T1; psychological detachment at T2 on spillover at T1) were estimated. Furthermore, cross-lagged paths were controlled by regressing covariates (i.e., gender, tenure, and caregiving at home) on all variables at T1 and T2.

**Figure 1:**
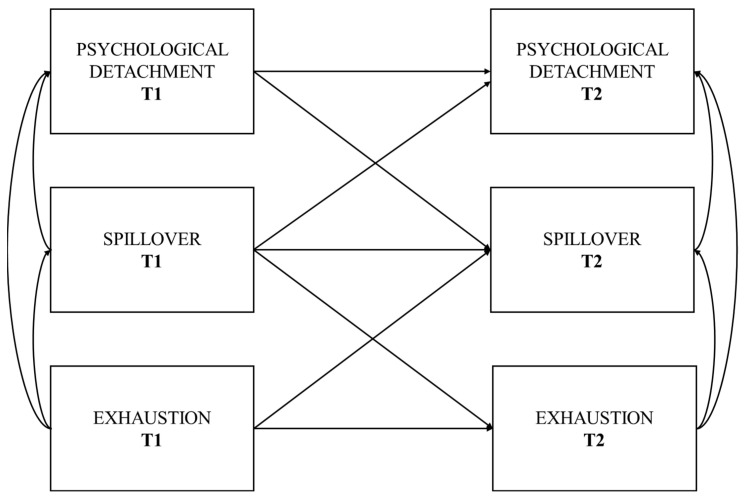
The conceptual model hypothesized in the present study. Notes: T1 (“Time 1”) indicates the initial measurement, while T2 (“Time 2”) indicates the follow-up measurement.

Practically, we implemented our hypothesized model (see Figure 1) in several steps. First, we assessed the validity of the hypothesized measurement model and its invariance across Time 1 (T1) and Time 2 (T2). To be sure, we built a measurement model including all three variables (i.e., psychological detachment, spillover, and exhaustion) at both T1 and T2, as latent factors with loadings of these specific indicators. In this measurement model, all latent variables were allowed to covary, and all residuals of observed indicators at T1 were allowed to correlate with their counterparts at T2. According to suggested procedures for testing measurement invariance (Byrne, 2013), we tested the model invariance across T1 and T2 at increasingly stringent levels: (1) configural invariance (i.e., no equality constraints are imposed; M1); (2) metric invariance (i.e., factor loading equality constraints are specified; M2); (3) scalar invariance (i.e., equality constraints on intercepts are specified; M3) and (4) strict invariance (i.e., equality constraints on residuals are specified; M4).

Once longitudinal measurement invariance was supported, we then tested our theoretical model by estimating and comparing four nested models: (1) a partial mediation model with the direct link from psychological detachment at T1 to exhaustion at T2, covariates and reciprocal relationships among variables (M1), (2) a partial mediation model with all non-significant effects of covariates fixed to be zero (M2), (3) a partial mediation model with all non-significant reciprocal regression paths fixed to be zero (M3), (4) the hypothesized full mediation model with the direct link from psychological detachment at T1 to exhaustion at T2 fixed to be zero (M4). We compared M3 and M4 to test whether the relationship between psychological detachment and exhaustion was fully or partially mediated by spillover.

### 2.4. Statistical Analyses

We used structural equation modeling (SEM) to evaluate the statistical model using the maximum likelihood (ML) estimator in the Mplus software program, version 8.1 (Muthén & Muthén, 2017). Regarding longitudinal measurement invariance, we employed a latent variable approach because it provides more reliable estimates by taking into account measurement errors (Byrne, 2013). At each step of invariance (i.e., configural, metric, scalar; strict), the model’s goodness of fit was evaluated with (1) the chi-square statistic (χ^2^; Byrne, 2013), (2) values of CFI higher than 0.90, (3) RMSEA values lower than 0.08 (Browne & Cudeck, 1992), and (4) SRMR values lower than 0.08 (Hu & Bentler, 1999). Measurement invariance was assessed through model differences in the models’ chi-square statistics (Δχ^2^), which should not be significant (Byrne, 2013). Since the chi-square statistic is sensitive to sample size, current recommendations suggest using multiple fit statistics to assess model fit (Byrne, 2013). Therefore, we assessed measurement invariance at each step also through the comparative fit index (ΔCFI) with values lower than −0.01, paired with changes in RMSEA of 0.015 and SRMR of 0.030 (for metric invariance) or 0.015 (for scalar and strict invariance) (Putnick & Bornstein, 2016).

Following this, the main hypotheses were tested via path analysis within the structural equation modeling (SEM) framework. Again, we assessed the appropriateness of each nested model (i.e., M1-M4) and tested whether there were significant differences between them (Byrne, 2013). To be sure, from the previous model to the next, structural paths were retained only if they were significant (*p* < 0.05), and we evaluated whether significant changes in Δχ^2^, as well as a deterioration of fit indices, occurred. Mediated effects were calculated using the procedures outlined by D. P. MacKinnon et al. (2002). The values for the upper and lower confidence intervals (CI) for indirect effects were tested with 1000 replications (D. MacKinnon, 2012).

## 3. Results

### 3.1. Zero-Order Correlations

All variables were significantly correlated in the expected direction (see Table 1), both cross-sectionally and longitudinally. Specifically, concurrent correlations at T1 were significant at *p* < 0.001 and ranged between |0.28| (psychological detachment with exhaustion) and |0.59| (spillover with exhaustion). Consistently, correlations ranged between |0.31| (psychological detachment with exhaustion) and |0.64| (psychological detachment with spillover) at time 2. Longitudinal correlations were also significant at *p* < 0.001 and ranged between |0.25| (exhaustion at T1 with psychological detachment at T2) and |0.62| (spillover at T1 with spillover at T2).

### 3.2. Structural Equation Analyses

#### 3.2.1. Measurement Models

As displayed in Table 2, all measurement models—M1 (configural model), M2 (metric model), M3 (scalar model), and M4 (strict model)—showed a reasonable fit to the data. Specifically, the configural model (M1) yielded good indices (χ^2^ = 408.160, df = 225, RMSEA = 0.056, CFI = 0.959, SRMR = 0.058), indicating that the hypothesized factor structure was acceptable at both time points without imposing any constraints. When factor loadings were constrained to be equal across time (metric invariance, M2), model fit remained adequate (χ^2^ = 428.630, df = 234, RMSEA = 0.057, CFI = 0.957, SRMR = 0.061), and the change in fit indices from M1 to M2 (Δχ^2^ = 20.468, Δdf = 9, *p* < 0.01; ΔCFI = −0.002; ΔSRMR = 0. 003) was within recommended thresholds, supporting metric invariance. Further constraints on intercepts (scalar invariance, M3: χ^2^ = 451.430, df = 245, RMSEA = 0.057, CFI = 0.954, SRMR = 0.062) resulted in minor and acceptable differences compared to M2 (Δχ^2^ = 22.805, Δdf = 11, *p* < 0.01; ΔCFI = −0.003; ΔSRMR = 0.001). Finally, strict invariance was tested by constraining residual variances to be equal across time (M4: χ^2^ = 500.490, df = 257, RMSEA = 0.060, CFI = 0.946, SRMR = 0.067). Again, the changes in fit from the previous model (Δχ^2^ = 49.060, Δdf = 12, *p* < 0.001; ΔCFI = −0.008; ΔSRMR = 0.005) remained within the conservative cutoffs for invariance (Byrne, 2013; Putnick & Bornstein, 2016). Altogether, these results provide evidence for longitudinal measurement invariance up to the strict level, indicating that the psychological constructs under investigation (i.e., psychological detachment, spillover, and exhaustion) were assessed equivalently across T1 and T2. Furthermore, all standardized factor loadings were statistically significant (*p* < 0.001) and above the acceptable threshold of 0.30 (ranging from 0.58 to 0.95 at T1 and from 0.67 to 0.95 at T2), confirming the appropriateness of each item as an indicator of the hypothesized latent dimensions.

#### 3.2.2. Structural Models and Mediation Analyses

**Figure 2:**
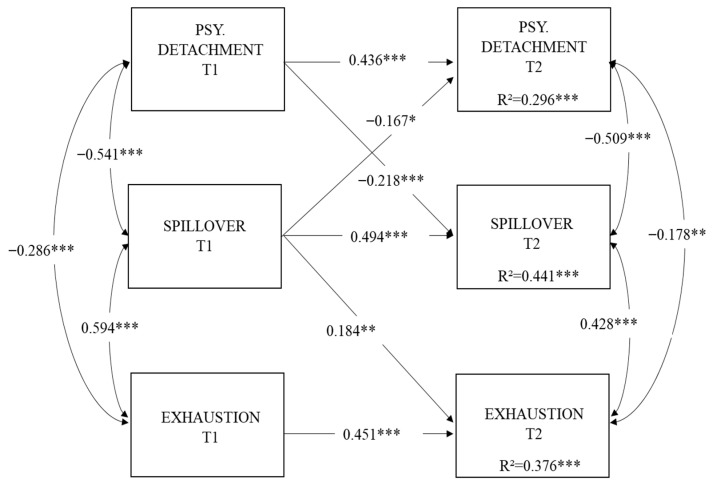
The hypothesized model with standardized estimates. Notes. This figure represents the hypothesized full mediation model in which non-significant paths and covariates are omitted for the sake of clarity. The effects of covariates, along with details about the parameter estimates, are reported in the text. Psy.Detachment = psychological detachment; * *p* < 0.05 ** *p* < 0.01; *** *p* < 0.001.

The hypothesized full mediation model (M4) demonstrated an excellent fit to the data, as indicated by the aforementioned criteria (χ2 = 16.140, df = 17, *p* > 0.05; CFI = 1.000; RMSEA [95% CI] = 0.000 [0.000, 0.054]; SRMR = 0.038). In accordance with our hypotheses (see Figure 2), (H1) psychological detachment at T1 predicted lower spillover at T2 (β = −0.218, SE = 0.053, *p* < 0.001), and (H2) spillover at T1 was significantly associated with higher levels of exhaustion at T2 (β = 0.184, SE = 0.062, *p* < 0.001). Then, we tested whether the longitudinal relationship between psychological detachment at T1 and exhaustion at T2 was mediated by spillover. The resulting unstandardized indirect effect was 0.053 (*p* < 0.05), and the associated bootstrapped CI did not include zero (LLCI = −0.106; ULCI = −0.016), therefore supporting mediation (H3). As such, the relationship between psychological detachment at T1 and exhaustion at T2 was fully mediated by spillover (D. Mackinnon, 2012). Regarding the expected reciprocal paths between variables, we confirmed one of our hypotheses. Specifically, we found that spillover at T1 was significantly and negatively associated with psychological detachment at T2 (β = −0.167, SE = 0.065, *p* < 0.05), thus supporting our hypothesis (H5). In contrast, reciprocal cross-lagged paths between exhaustion at T1 and spillover (β = 0.057, SE = 0.061, *p* > 0.05) and psychological detachment (β = −0.021, SE = 0.070, *p* > 0.05) at T2 were not significant. Therefore, exhaustion did not predict subsequent spillover or detachment, providing no support for H4 and H6. Finally, the regressive paths of covariates were kept in the model only when they resulted in significant effects. Compared to males, females showed higher levels of spillover both at T1 (β = 0.137, SE = 0.053, *p* < 0.01) and T2 (β = 0.131, SE = 0.042, *p* < 0.001) Moreover, females showed higher exhaustion both at T1(β = 0.197, SE = 0.059, *p* < 0.01) and T2 (β = 0.112, SE = 0.051, *p* < 0.05). Overall, the model explained a substantial amount of variance in spillover at T2 (44.1%) and exhaustion at T2 (37.6%).

## 4. Discussion

Grounded in the Conservation of Resources theory (Hobfoll, 1989) and the Stressor–Recovery model (Sonnentag & Fritz, 2007), the present cross-lagged study, with a 2-year time lag, explored the interplay between psychological detachment and exhaustion, positing spillover as a mediating mechanism leading to impaired well-being. Specifically, we examined the direct relationship between psychological detachment, spillover, and exhaustion, as well as the reverse causal model; namely, whether individual strain levels may lead to a decreased recourse to recovery strategies (Sonnentag, 2018). Overall, most of our hypotheses were confirmed. We found that psychological detachment was negatively related to spillover, resulting in decreased vulnerability to exhaustion. As such, people who managed to detach themselves from work-related events at the end of the shift were less likely to think back to those emotionally triggering episodes once at home and to feel exhausted in the long run. These results corroborate prior studies on this topic (e.g., Sianoja et al., 2018; Sonnentag et al., 2010), showing the beneficial role of mentally disengaging from work in fostering individuals’ well-being and reducing their individual level of exhaustion. Moreover, our findings extend the literature on recovery by identifying spillover as a psychological mechanism underlying the relationship between psychological detachment and exhaustion. Indeed, we found that spillover totally mediates the aforementioned direct path, as detachment prevents the rethinking of work-related events from spilling over into one’s private life and emotionally draining the individuals’ resources (Hobfoll, 1989) by their active mental recall. Additionally, although no previous studies have posited spillover as a mediating mechanism between psychological detachment and exhaustion, our results align with prior research on this topic. For instance, Sonnentag and Binnewies (2013) showed that psychological detachment from work attenuates negative spillover during non-work hours. In contrast, Hall et al. (2010) found that the level of exhaustion was linked to the spillover of job demands into the private sphere, resulting in higher work–family conflict. On the other hand, through the explicit testing of the reverse relationships (exhaustion–spillover–detachment), we did not find a significant association either between exhaustion and spillover or between exhaustion and psychological detachment.

This pattern suggests that exhaustion, although a major indicator of strain, does not necessarily trigger cognitive or emotional spillover into non-work life, nor does it automatically undermine one’s capacity to mentally disengage from work. From a theoretical perspective, these null findings refine the Stressor–Detachment Model by indicating that recovery processes may not simply mirror strain accumulation. Rather, detachment and spillover might depend on distinct self-regulatory and boundary management capacities that operate relatively independently from momentary levels of exhaustion (Sonnentag, 2018). Furthermore, the long time lag adopted in this study (two years) may have diluted short-term reciprocal effects between exhaustion and detachment, which could be more detectable over shorter intervals. Overall, these results support a more dynamic view of recovery, where exhaustion and detachment interact over time but do not necessarily exert symmetrical causal influences. These findings contribute to the literature on the rarely addressed reverse causation between exhaustion and detachment, which revealed conflicting findings (Sonnentag et al., 2010, 2014) and emphasized the necessity for additional exploration of this relationship over time (Sonnentag, 2018). Nevertheless, we found an interesting result concerning the reverse relationship between spillover and psychological detachment. Indeed, people who tend to keep thinking about work-related topics even when at home, while simultaneously facing difficulties in engaging in personal life, experience greater challenges in psychologically detaching from work. This result aligns with earlier studies (see Cropley & Zijlstra, 2011), illustrating how spillover significantly impacts individuals’ ability to unwind successfully after a demanding workday. Additionally, it highlights the extent to which recovery processes depend on an individual’s ability (or inability) to disentangle themselves from job demands and associated thoughts (Cropley et al., 2006; Sonnentag et al., 2008).

Finally, a socio-demographic variable was found to be significant. Specifically, our results showed that women presented higher scores in both spillover and exhaustion levels at T1 and T2 than men. This finding corroborates prior research on this issue, showing women’s greater vulnerability to stress and exhaustion (e.g., Zender & Olshansky, 2009; Purvanova & Muros, 2010). Previous studies have suggested that this pattern may reflect both contextual stressors and gendered coping tendencies. In healthcare settings, women are often exposed to higher emotional and relational demands. They are more frequently engaged in direct caregiving roles, which require sustained empathic involvement and emotional regulation. In our study, we controlled for caregiving at home, which had no significant impact. However, gendered social roles outside work—such as the greater burden of household and family responsibilities—may exacerbate work–family conflicts and hinder recovery opportunities (Bianchi & Schonfeld, 2016). Moreover, women may rely more on emotion-focused coping strategies, which can intensify emotional strain over time. Recent longitudinal evidence by Bernstrøm et al. (2025) shows that after-hours work and extended schedules increase burnout and reduce psychological detachment by intensifying work–home conflict. These contextual factors may also interact with gendered experiences and coping tendencies, potentially amplifying women’s exposure to chronic demands and reducing their opportunities for recovery.

### 4.1. Theoretical and Practical Implications

The present study offers several theoretical contributions to the Stressor–Detachment Model and the Conservation of Resources (COR) theory. First, it advances the Stressor–Detachment Model by identifying spillover as a core psychological mechanism explaining how insufficient detachment translates into exhaustion. While previous research has emphasized the outcomes of detachment, our findings clarify how detachment exerts its protective role by limiting the cross-domain transmission of work-related affective activation into non-work time. This specification refines the Stressor–Detachment Model by introducing a mediating process that links recovery experiences to well-being outcomes.

Second, the study extends the COR theory by demonstrating that detachment functions as a resource-preserving strategy that actively interrupts resource loss spirals. Spillover represents the pathway through which resource depletion is perpetuated across domains, whereas detachment serves as a cognitive gatekeeper preventing this cycle. In this sense, our findings illuminate the micro-level processes through which the COR principle of resource loss and gain operates in daily recovery dynamics.

Third, by testing reverse causal effects, the study adds nuance to both theoretical frameworks, showing that exhaustion does not automatically erode recovery capacities over time. This suggests that recovery and strain processes follow partially independent trajectories, calling for a more dynamic and reciprocal understanding of stress and recovery models.

Ultimately, the observed gender differences underscore the need for a contextualized and gender-sensitive extension of recovery theories, incorporating socio-demographic and occupational factors into models that have traditionally focused on individual processes.

From a practical standpoint, our research suggests applicational avenues for promoting employees’ well-being and healthy workplaces. Specifically, healthcare contexts should foster a work environment that supports and stimulates employees, promoting interventions at both individual and organizational levels. According to our study, employees should be encouraged to mentally disengage from work once they are at home, allowing for time to focus on physical and emotional recovery. Indeed, healthcare professionals are particularly exposed to adverse well-being outcomes (L. S. Meredith et al., 2022), mainly due to the emotional burden associated with their work; hence, it is essential to raise employees’ awareness of the potential beneficial effects of detaching from one’s job, especially after a demanding and emotionally challenging working day (Dall’Ora et al., 2015). Along with this, since “mentally switching off” from work can be trained (Hahn et al., 2011), it would be important for the healthcare sector to coach managers on practices that facilitate employees’ mastery of greater psychological detachment, such as giving training and coaching (Locke & Latham, 2015) in the critical handling of emotional demands. At the same time, managers could promote recovery strategies directly during the working day, such as by allowing micro-breaks when needed (Wang et al., 2022), as well as stabilizing shifts, reducing workload, and ensuring peer networking (Cohen et al., 2023). Moreover, it might be helpful to integrate expert personnel within the staff (e.g., psychologists) who can support employees in managing stress and taking charge of the psychological–emotional management of families and patients by enhancing interdisciplinary intervention. On the other hand, employees need to be supported in constructively defining their personal and work boundaries according to one’s need (Ashforth et al., 2000), as well as becoming aware of some helpful recovery strategies to mitigate the spillover of work-related events into the family context when the workday ends (e.g., engaging in hobbies, social activities or in physical exercise). Indeed, learning to recognize and communicate one’s need for recovery proves to be a highly protective personal resource, especially in work contexts where multiple job demands must be managed. Taken together, these types of interventions must fit into an organizational climate that promotes a culture of self-care, paying attention to employees’ needs, and being aware of the material and human resources to meet them.

### 4.2. Limitations and Future Research

This study has some limitations that should be addressed. First, the study relied only on online self-report measures, which may entail potential biases in response accuracy and increase the risk of inferential errors. Although the two-wave design helps to mitigate concerns about common method bias and temporal ambiguity, self-report data still depend on participants’ subjective perceptions and may be influenced by memory or attributional biases. However, psychological detachment, spillover, and exhaustion are inherently subjective experiences that can be meaningfully assessed only through personal introspection (Conway & Lance, 2010; Podsakoff et al., 2012). Nevertheless, future studies should combine self-report data with multiple source indicators (e.g., a manager in the work domain, the partner in the family domain) to enhance the validity of inferences regarding the observed relationships.

Second, the limited temporal scope of the present study represents an important methodological constraint. Although our two-wave (half-longitudinal) design allowed for testing lagged relationships over a meaningful interval, aligning with our research focus on between-person differences and long-term associations among detachment, spillover, and exhaustion, the two-year spacing between waves prevented the examination of short-term, non-linear, or reciprocal dynamics among these variables. Moreover, the autoregressive cross-lagged model relies on the assumption of stationarity, meaning that structural relations remain stable across measurement occasions. This assumption may be challenged by the fact that psychological detachment is a fluctuating experience that can vary over shorter intervals. Future research should therefore employ full longitudinal designs with multiple and more frequent measurement waves (e.g., quarterly assessments) to test whether these relationships remain stable or vary over time. Approaches such as growth curve modeling or multilevel longitudinal models would also allow researchers to model both stable between-person patterns and potential time-varying within-person changes, thus providing a more dynamic and fine-grained understanding of recovery processes.

Fourth, in our study, we focused exclusively on psychological detachment as a recovery strategy. Although detachment represents a core mechanism in the recovery process, which has received the most research attention (Agolli & Holtz, 2023; Sonnentag et al., 2022), other recovery experiences—such as relaxation, mastery, or control (Sonnentag & Fritz, 2007)—may also contribute to psychological restoration and could interact with detachment in complex ways. Further investigations should explore whether relaxation, mastery, or control exerts similar, distinct, or even synergistic effects within the same mediational framework.

Finally, our study did not specifically address contextual factors that may account for variations in our study variables. Recent longitudinal evidence by Bernstrøm et al. (2025) shows that after-hours work and extended schedules increase burnout and reduce psychological detachment by intensifying work–home conflict. Future studies should therefore integrate contextual factors—such as working hours, shift patterns, and organizational norms regarding after-hours availability—that may reduce employees’ opportunities to recover over time.

## 5. Conclusions

This study examined how psychological detachment prevents exhaustion in healthcare workers over time, identifying reduced work–home spillover as a key mediating mechanism. Using a two-wave longitudinal design, we found that detachment at the end of the workday reduces the mental and emotional intrusion of work into private life, which in turn lowers exhaustion levels over time. Notably, spillover fully mediated the detachment–exhaustion relationship, positioning detachment as a cognitive gatekeeper that regulates resource loss. We also tested reverse pathways and found that while spillover predicted reduced detachment over time, exhaustion did not significantly affect either spillover or detachment. These findings refine the Stressor–Detachment Model and align with the Conservation of Resources theory, emphasizing the role of detachment in preventing the cycle of resource depletion. This research offers valuable implications for supporting employees in emotionally demanding professions, such as healthcare professionals, highlighting the importance of protecting the recovery process and experiences to sustain long-term well-being. All in all, this study advances understanding of recovery processes by integrating the Stressor–Detachment Model and the Conservation of Resources theory, identifying spillover as a key mechanism linking detachment and exhaustion. The findings underscore the importance of promoting conditions that enable employees to disengage from work mentally. In line with the study’s limitations, future research should adopt multi-method, multi-wave approaches and include additional recovery experiences to capture the dynamic nature of recovery over time.

## Figures and Tables

**Table 1:** Descriptive statistics and zero-order correlations.

	M	SD	(1)	(2)	(3)	(4)	(5)	(6)
(1) Psychological Detachment T1	3.99	1.56	(0.91)					
(2) Spillover T1	2.68	0.65	−0.54	(0.79)				
(3) Exhaustion T1	4.03	1.66	−0.28	0.59	(0.92)			
(4) Psychological Detachment T2	3.92	1.59	0.54	−0.40	−0.25	(0.94)		
(5) Spillover T2	2.64	0.82	−0.49	0.62	0.42	−0.64	(0.85)	
(6) Exhaustion T2	4.12	1.64	−0.27	0.46	0.59	−0.31	0.58	(0.91)

Notes. All correlations were significant at *p* < 0.001; M = mean; SD = standard deviation. Coefficient alpha reliability estimates are presented in brackets along the diagonal.

**Table 2:** Results of Tests for Measurement Invariance across T1 and T2.

Model	χ^2^	*df*	RMSEA [CI 95%]	CFI	SRMR	ΔM	Δχ^2^ (Δdf)	ΔCFI	ΔRMSEA	ΔSRMR
M1	408.160 ***	225	0.056 [0.047 0.065]	0.959	0.058	−	−	−	−	−
M2	428.630 ***	234	0.057 [0.048 0.65]	0.957	0.061	M1–M2	20.468 (9) **	−0.002	0.001	0.003
M3	451.430 ***	245	0.057 [0.049 0.065]	0.954	0.062	M2–M3	22.805 (11) **	−0.003	0.000	0.001
M4	500.490 ***	257	0.060 [0.053 0.068]	0.946	0.067	M2–M3	49.060 (12) ***	−0.008	0.003	0.005

Notes. Increasingly restrictive models (configural, metric, scalar) are compared to assess whether the measurement structure is stable across waves. ** *p* < 0.01; *** *p* < 0.001; At each step the prior model served as the baseline against which the subsequent specified model was compared in the sequence of invariance tests, all earlier constraints remained in place; M1 = configural model; M2 = model with metric invariance for each latent dimension; M3 = model with scalar invariance for each latent dimension; M4 = model with strict invariance for each latent dimension; χ2 = chi-square statistic; df = degrees of freedom; RMSEA = Root Mean-Square Error of Approximation; CFI = Comparative Fit Index; SRMR = Standardized Root Mean Square Residual.

## Data Availability

The data are available upon request from the first author.
